# Hyperostosis fronto-parietalis – radiology mimic of metastasis: A case report

**DOI:** 10.1016/j.radcr.2021.05.036

**Published:** 2021-06-21

**Authors:** Szeyi Lai, Neeru Tomer

**Affiliations:** Department of Radiology, Darlington Memorial Hospital, Hollyhurst Rd, Darlington, DL3 6HX, UK

**Keywords:** Hyperostosis frontalis interna, Hyperostosis fronto-parietalis, Bone metastasis, Computed tomography

## Abstract

Hyperostosis frontalis interna (HFI) is a benign entity manifested by bony overgrowth in the frontal endocranial surface. It is most commonly reported incidentally among postmenopausal elderly women. Tracer uptake appearances of HFI can vary on planar bone scans, enabling it to be easily confounded with bone metastases. We report a case of HFI in a 69-year-old postmenopausal female with treated left breast cancer detected on bone scintigraphy, with subsequent confirmation by computed tomography. Our case highlights the importance of having awareness of HFI and its key pattern findings to avoid mistaking it for pathology, and to recognise the use of computed tomography and hybrid fusion imaging techniques as reliable diagnostic tools for HFI.

## Introduction

Hyperostosis frontalis interna (HFI) is characterized by irregular overgrowth of bony tissue in the frontal bone inner table. The condition is often found incidentally among postmenopausal elderly women and is typically benign. However, marked calvarial thickening can cause compression of the frontal lobes. Tracer uptake appearances of HFI on bone scintigraphy studies can be variable, enabling it to be easily confounded with bone metastases. Recognition of this entity and its pattern findings is important to avoid mistaking it for pathology. In addition to the use of computed tomography (CT) as a reliable diagnostic tool for HFI, hybrid fusion imaging techniques can add value as a useful adjunct to guide identification and characterization of HFI, as well as to discriminate between benign and malignant processes.

## Case presentation

**Fig. 1:**
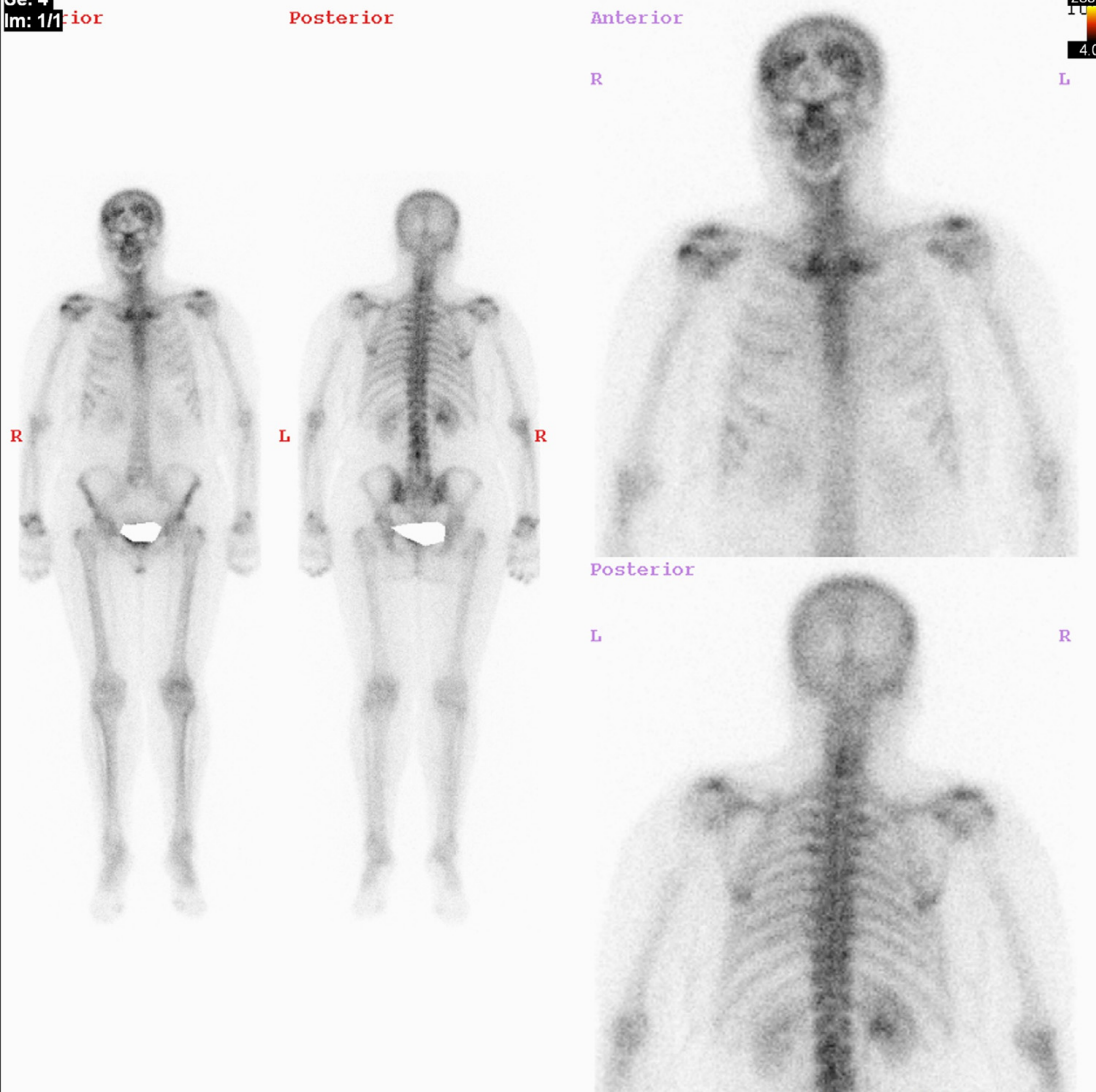
99mTc-methylene diphosphonate planar bone scan reveal asymmetrical abnormal increased tracer uptake involving the bilateral fronto-parietal regions of the skull. Tracer uptake in the remaining skeleton were within normal limits.

**Fig. 2:**
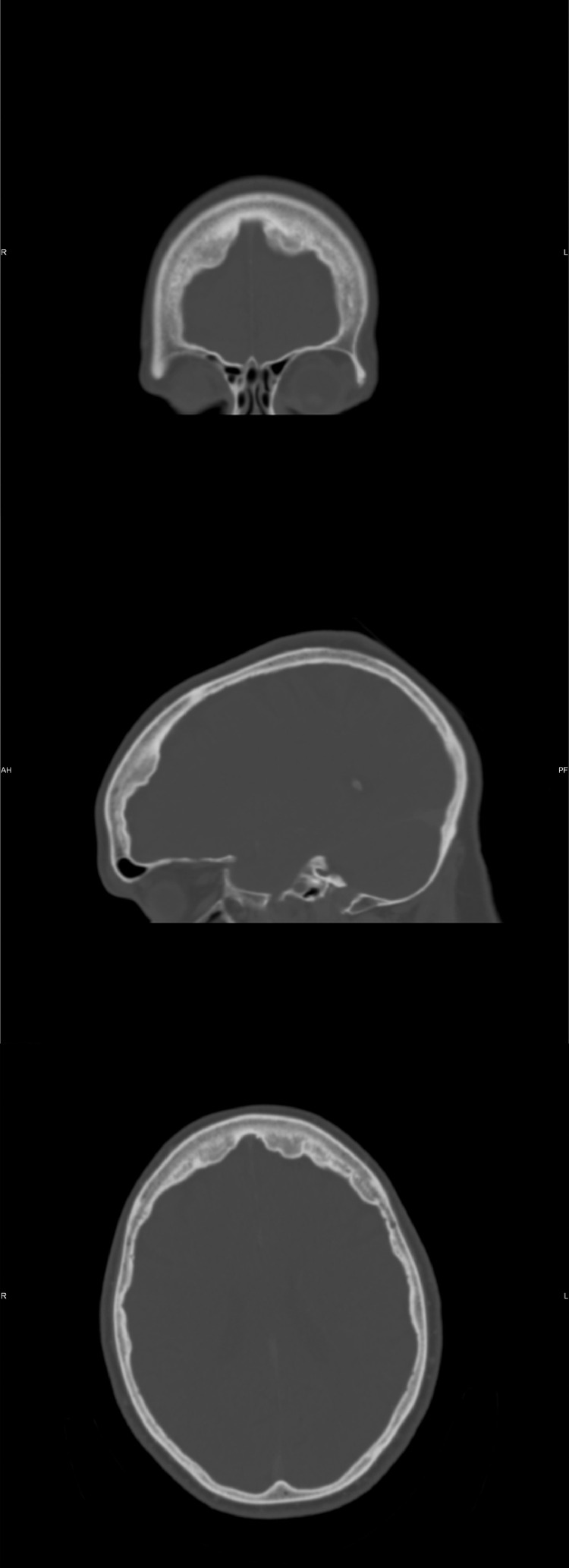
Sagittal, axial and coronal CT images of the skull show irregular, nodular thickening of the frontal bone inner table extending to involve the parietal bones, with sparing of the midline and outer calvarial surface.

The 69-year-old female patient with a background of treated left breast cancer in 2012 presented with a history of gradual, progressive left-sided chest wall and breast pain. An initial mammography and ultrasound of both breasts were carried out which demonstrated BI-RADS 1 findings. Bone scintigraphy was performed to exclude skeletal involvement. The results of this study revealed asymmetrical abnormal increased tracer uptake involving the bilateral fronto-parietal regions of the skull [Fig. 1]. Tracer uptake in the remaining skeleton were within normal limits for her age. To further evaluate this lesion a CT examination of the skull was performed. Findings showed irregular thickening of the frontal bone inner table extending to involve the parietal bones [Fig. 2]. The midline was spared and periosteum and cortical bone were unaffected. Overall features were consistent with hyperostosis fronto-parietalis. No definite evidence of lytic or sclerotic deposits was noted.

Origin: Department of Radiology, Darlington Memorial Hospital, Hollyhurst Rd, Darlington, United Kingdom.

## Discussion

HFI is a benign entity manifested by irregular overgrowth of bony tissue in the inner table of the frontal bone, which is typically bilateral and symmetrical. Literature has shown HFI to be an age- and sex-related phenomena [5], with the highest incidence found in elderly postmenopausal women, 40% to >60% [1,^7^,9]. The aetiopathogenesis of HFI remains ambiguous, however current hypotheses implicate hormonal imbalance with prolonged oestrogen stimulation proposed [8,12].

The condition is often found incidentally and is generally of no clinical significance [9]. However, marked HFI can cause compression of the frontal lobes [3]. Numerous case reports have shown HFI to be associated with conditions including frontal headaches, psycho-neurosis, obesity and diabetes [4,^6^,^14^,16].

On CT imaging, HFI has unique characteristics which help to distinguish it from other endocranial bony overgrowths including (1) bifrontal symmetry; (2) distinct boundaries without crossing the sagittal sinus area medially; (3) expansion over the frontal endocranial surfaces; (4) continuity with the inner table and diploe [2,^10^,13].

Utilizing plain radiographs, Moore [14] grouped HFI under the broad category of metabolic craniopathy, which included nebula frontalis, hyperostosis calvaria diffusa, and hyperostosis fronto-parietalis, named according to the lesion location. Hershkovitz et al. [8] systematically categorized HFI into types A-E, based on various morphologic characteristics. In addition to this, Hershkovitz et al. [8] demonstrated the use of CT to be a more reliable diagnostic tool as compared to conventional radiographs, with only 44.1% of HFI Type B cases accurately identified via plain radiograms vs. 93.3% by CT. May et al. [13] showed that a CT-based system using a 3D volume rendering viewing protocol and CT characteristics can reliably identify and characterize HFI with a 91.3% positive predictive value. Having said that, a limitation of the method was the inadequacy in identifying very early stages of HFI.

Increased tracer uptake in thickened areas of the frontal bone is a frequent finding on bone scintigraphy studies [15]. Increased osteoblastic activity on 18F-NaF positron emission tomography-computed tomography (PET/CT) has been reported without any demonstrable radiographic abnormality which suggests this to be an early marker of HFI [19]. Given tracer uptake appearances can be variable on planar or maximum intensity projection images, this can easily be confounded with bone metastases [11]. In these cases, evaluation with hybrid fusion imaging such as PET/CT or single-photon emission computed tomography/computed tomography can be valuable by identifying and characterizing tracer uptake in areas of frontal bone overgrowth.

## Conclusion

Taking into consideration the increasing prevalence and severity of HFI over time [2,8], the awareness of key pattern findings on CT studies is pivotal during the diagnostic process in order to avoid misinterpretation and minimize the reporting of false positive metastases [17,18]. Utilizing volume rendering with a CT 3-scale classification has been shown to be a reliable and valid tool for radiological diagnosis of HFI [13], avoiding undesirable diagnostic delays and unnecessary investigations.

In some cases, the use of hybrid fusion imaging techniques including PET/CT and single-photon emission computed tomography/computed tomography can add value by demonstrating specific findings, assisting in the identification and classification of HFI. It is important to recognise these techniques as a useful adjunct in the evaluation and discrimination between benign and malignant processes.

## Learning points

• HFI is a benign entity manifested by irregular overgrowth of bony tissue in the frontal bone endocranial surface and is most commonly reported as an incidental finding among postmenopausal elderly women.
• Tracer uptake appearances of HFI can vary on planar bone scans, enabling it to be easily confounded with bone metastases. In view of this, it is important to be aware of HFI and its key pattern findings to avoid mistaking it for pathology.
• Utilising volume rendering with a CT 3-scale classification has been shown to be a reliable and valid tool for radiological diagnosis of HFI. Hybrid fusion imaging techniques can add value as a useful adjunct to guide identification and characterization of HFI, as well as to discriminate between benign and malignant processes.

## Patient consent

Written informed consent for publication was obtained from the patient.

## Ethical approval

This article does not contain any studies with human participants or animals performed by any of the authors.
